# Extensive *in silico* analysis of Mimivirus coded Rab GTPase homolog suggests a possible role in virion membrane biogenesis

**DOI:** 10.3389/fmicb.2015.00929

**Published:** 2015-09-15

**Authors:** Amrutraj Zade, Malavi Sengupta, Kiran Kondabagil

**Affiliations:** Department of Biosciences and Bioengineering, Indian Institute of Technology-BombayMumbai, India

**Keywords:** NCLDV, APMV, Rab GTPase, subfamily specific (SF) region, membrane acquisition, virus assembly

## Abstract

Rab GTPases are the key regulators of intracellular membrane trafficking in eukaryotes. Many viruses and intracellular bacterial pathogens have evolved to hijack the host Rab GTPase functions, mainly through activators and effector proteins, for their benefit. *Acanthamoeba polyphaga mimivirus* (APMV) is one of the largest viruses and belongs to the monophyletic clade of nucleo-cytoplasmic large DNA viruses (NCLDV). The inner membrane lining is integral to the APMV virion structure. APMV assembly involves extensive host membrane modifications, like vesicle budding and fusion, leading to the formation of a membrane sheet that is incorporated into the virion. Intriguingly, APMV and all group I members of the *Mimiviridae* family code for a putative Rab GTPase protein. APMV is the first reported virus to code for a Rab GTPase (encoded by R214 gene). Our thorough *in silico* analysis of the subfamily specific (SF) region of *Mimiviridae* Rab GTPase sequences suggests that they are related to Rab5, a member of the group II Rab GTPases, of lower eukaryotes. Because of their high divergence from the existing three isoforms, A, B, and C of the Rab5-family, we suggest that *Mimiviridae* Rabs constitute a new isoform, Rab5D. Phylogenetic analysis indicated probable horizontal acquisition from a lower eukaryotic ancestor followed by selection and divergence. Furthermore, interaction network analysis suggests that vps34 (a Class III PI3K homolog, coded by APMV L615), Atg-8 and dynamin (host proteins) are recruited by APMV Rab GTPase during capsid assembly. Based on these observations, we hypothesize that APMV Rab plays a role in the acquisition of inner membrane during virion assembly.

## Introduction

With a particle size of about 750 nm, *Acanthamoeba polyphaga mimivirus* (APMV) is one of the largest viruses known so far (La Scola et al., 2003; Claverie et al., 2006). APMV belongs to the monophyletic clade of large eukaryotic DNA viruses known as the nucleo-cytoplasmic large DNA viruses (NCLDV) (Iyer et al., 2001). In the mature APMV particle, its 1.2 Mbp linear genome is encapsidated within the icosahedral capsid that is underlined by a lipid bilayer (Xiao et al., 2005). Acquisition of inner viral membrane is a critical step during the capsid assembly and is poorly understood.

APMV is a cytoplasmically replicating virus that carries out viral genome replication transcription, protein synthesis and the subsequent stages of particle formation and virus budding in the giant cytosplasmic structures known as viral factories. Viral factories are formed at 4 h post infection (PI) and the budding of new viral particles has been observed at around 8 h PI (Suzan-Monti et al., 2007; Zauberman et al., 2008). The membrane biogenesis of APMV is initiated from the host cytoplasmic membrane cisternae at around 7.5 h PI (Zauberman et al., 2008; Mutsafi et al., 2013). Regular budding of ~70 nm vesicles from the cellular cisternae have been observed around the viral factories at earlier PI times (Mutsafi et al., 2013). Continuous fusion of smaller vesicles leads to the formation of multivesicular bodies followed by its fissure to form large membrane sheets that are incorporated into the viral progenies (Mutsafi et al., 2013). The major capsid protein L425, a homolog of vaccinia virus D13, acts as a scaffolding protein and initiates capsid assembly above the membrane sheet. A consistent membrane overhang that prevents the premature closure of the capsid is trimmed off upon completion of the capsid assembly, leaving a ~20 nm nonvertex transient opening for genome packaging (Mutsafi et al., 2013).

Rab GTPases, a subfamily of small GTP binding proteins within the Ras superfamily, are the key regulators of membrane trafficking (Bourne et al., 1990). Rab GTPase functions by alternating between two states; a GTP-bound active state in which it interacts with effector proteins and a GDP-bound inactive state in which it interacts with proteins like Rab escort protein (REP) and GDP dissociation inhibitor (GDI) (Lee et al., 2009). Functionally divergent Rab GTPases are involved in budding and scission of membrane vesicles from donor organelles, their transport along the actin and microtubules, association with target membrane through tethering complex, and finally their fusion with recipient organelle (Zerial and McBride, 2001). Many intracellular pathogens reside in the vacuoles and extensively modify its host-derived membrane by either recruiting or excluding surface Rab proteins with the help of pathogen proteins (Brumell and Scidmore, 2007; Kumar and Valdivia, 2009). Aside from *Legionella pneumophila*, which encode proteins that directly interact with Rab, other Rab-mediated mechanisms employed by bacterial pathogens remain poorly understood (Brumell and Scidmore, 2007).

Rab GTPases are the hallmarks of the eukaryotic endomembrane system that are found in prokaryotes. It is quite intriguing for a virus, which does not have a cellular structure, to code for this particular protein. In this report, we present a thorough sequence, structural, and phylogenetic analysis of Rab GTPases of *Mimiviridae* Group I viruses. Our results indicate that *Mimiviridae* Rabs belong to Rab5 family and were probably acquired horizontally from an early unicellular eukaryote. Further, sequence and structural comparisons suggest that *Mimiviridae* Rabs possess all the signature motifs of family Rab5, and have probably diverged to form a new isoform, 5D. Considering the above observations and the available APMV transcriptome data and membrane assembly insights (Legendre et al., 2011; Mutsafi et al., 2013), we speculate that the APMV Rab GTPase might play a role in the acquisition of inner membrane during capsid assembly.

## Materials and methods

### Retrieval of Rab sequences

The putative Rab GTPase amino acid sequences from *Mimiviridae* family viruses were retrieved from UniprotKB. The APMV R214 sequence was used as a query to search for homologous sequences in the nr (nonredundant) GenBank protein database. Sequence similarity searches were performed using the BLASTP application (http://blast.ncbi.nlm.nih.gov/Blast.cgi) with standard settings except the maximum target sequences menu under General Algorithm parameters was changed to 250 from 100. Redundant, unnamed and hypothetical sequences were removed before the alignment. A dataset for comparative analysis was prepared using all the annotated Rab GTPase isoforms from *Homo sapiens, Plasmodium falciparum, Caenorhabditis elegance, Drosophila melanogaster, Trichomonas vaginalis*, and *Saccharomyces cerevisiae*.

### Sequence analysis, conserved motif identification, and phylogenetic reconstruction

*Mimiviridae* Rab GTPase sequences were subjected to multiple sequence alignment using Clustal Omega (Sievers et al., 2011) with default settings. The alignment was subjected to the ESPript 3.0 (Gouet, 2003) and secondary structure was predicted using PSIPRED (McGuffin et al., 2000) and Jpred3 servers (Cole et al., 2008).

Phylogenetic reconstruction was done in MEGA6 (Molecular Evolutionary Genetics Analysis) program (Tamura et al., 2013). Sequences were aligned using ClustalW with BLOSUM62 as weight matrix. Neighbor-Joining (NJ) method based trees were generated with complete deletion and p-distance options employed in MEGA6. Trees were analyzed using Bootstrap method for 1000 iterations (Nei and Kumar, 2000). MEGA6 was also used for visual details and representation.

### Structure prediction and alignment

APMV R214 sequence was threaded to I-TASSER online server (Zhang, 2008) against the I-TASSER template database for homology based structural modeling. Structure with the best C-score of 0.59, was selected for further analysis. *Homo sapiens* Rab5b (HsRab5b) structure was retrieved from PDB database (2HEI). Predicted APMV Rab and HsRab5b structures were aligned using PyMol (DeLano, 2002).

### Interaction network

APMV Rab interaction network was prepared using the Cytoscape platform (Saito et al., 2012). *P. falciparum* was used as the source organism to generate APMV Rab interactome. *P. falciparum* Rab5B (*Pf* Rab5B) physical interaction network was retrieved from StringDB and homologous protein sequences were obtained from UniprotKB database. Interacting species were used to search for their homologs using multiple reciprocal BLASTP at NCBI (http://blast.ncbi.nlm.nih.gov/Blast.cgi) against either APMV or *Acanthamoeba castellanii str. Neff* sequence datasets. Orthologs with *E* < 0.05 were selected and used to build the physical interaction network.

## Results

### All members of the group I *Mimiviridae* family encode for Rab GTPase

**Figure 1:**
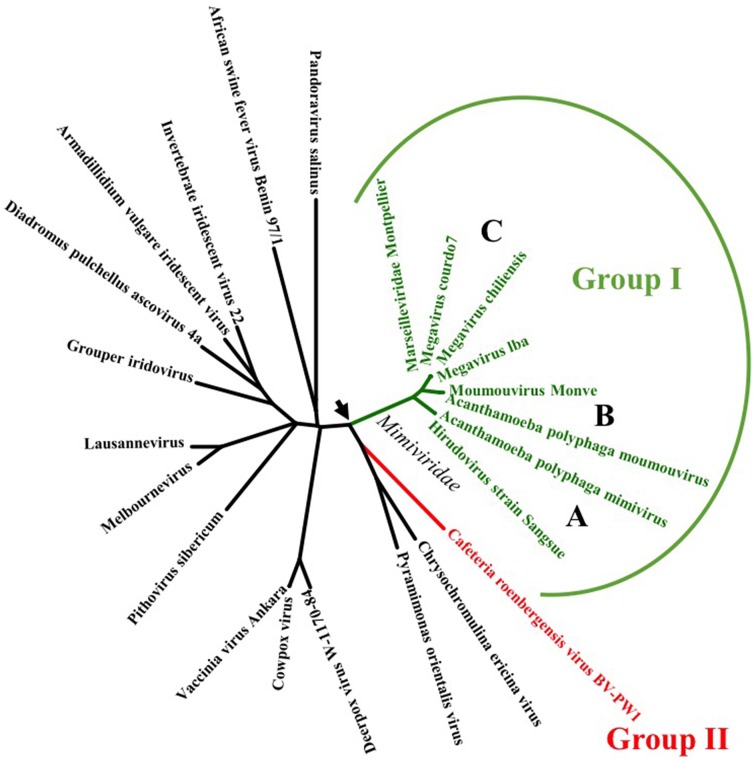
**Phylogenetic tree of DNA polymerase B from NCLDVs showing the probable acquisition of a Rab GTPase by an ancestor of ***Mimiviridae*** family**. **(A–C)** lineages of *Mimiviridae* group I viruses diverged from the group II sharing a common node which could be the point of acquisition of Rab gene.

A thorough BLASTP search of the NCBI GenBank database, with APMV R214 gene, that was annotated as a probable small GTPase protein (Raoult et al., 2004) as the query sequence against all members of the NCLDV superfamily, revealed that only Moumouvirus, Mamavirus, Lentille virus, Hirudovirus, Courdo virus, Samba virus, and Megavirus code for R214 homolog (Table 1). Interestingly, all seven viruses belong to the group I of *Mimiviridae* family of NCLDVs. A phylogenetic tree, constructed using the DNA polymerase B, with representative sequences from all NCLDVs, suggests that Rab GTPase was acquired by an ancestor of group I *Mimiviridae* lineages (Figure 1).

**Table 1:** **Rab GTPases in ***Mimiviridae*** family**.

**Lineage**	**Virus**	**Gene Name**	**UniProt ID**
A	*Acanthamoeba polyphaga mimivirus*	MIMI_R214	Q5UQ27
	*Samba virus*	N/A	W6GH08
	*Hirudovirus strain Sangsue*	HIRU_S752	V5L429
	*Acanthamoeba polyphaga lentillevirus*	R167	J3IZ12
	*Acanthamoeba polyphaga mamavirus*	MAMA_R280	G8ECS6
B	*Moumouvirus goulette*	glt_00705	M1NN89
	*Acanthamoeba polyphaga moumouvirus*	Moumou_00240	L7RBD8
	*Moumouvirus monve*	mv_L827	H2EFX7
C	*Megavirus chiliensis*	Mg314	G5CS79
	*Megavirus iba*	LBA_00299	L7Y3B3
	*Megavirus courdo11*	CE11_00318	K7YVK8
	*Megavirus courdo7*	c7_R350	H2EAJ3

### R214 gene product and its *Mimiviridae* group I homologs are Rab GTPases

**Figure 2:**
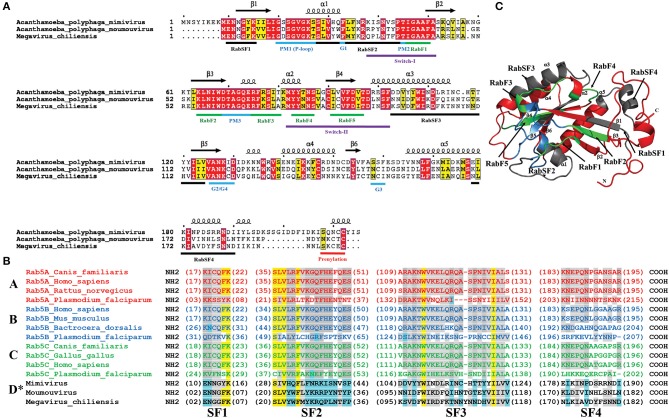
**A multiple sequence alignment of the amino acid sequences of ***Mimiviridae*** Rab GTPases. (A)** Signature motifs RabF, RabSF, and GTP binding, switch regions, and prenylation motif are highlighted in the alignment with green, black, blue, purple, and red lines, respectively. **(B)** Alignment of subfamily specific regions of Rab5 isoforms. The subfamily specific SF domains of *Mimiviridae* family and other annotated Rab5 isoforms, A, B, and C, are aligned to delineate *Mimiviridae* Rabs into a separate isoform Rab5D. Yellow color indicates strict conservation and gray color indicates conservation of a particular residue in lower eukaryotes and NCLDVs and, cyan color indicates the conserved residues specific to *Mimiviridae* family. ^*^Proposed new isoform of Rab5. **(C)** Predicted Mimivirus Rab GTPase structure. RabSF, RabF, and GTP binding regions in the predicted structure are denoted in red, blue, and yellow, respectively.

The signature Rab GTPase motifs, the PM1 motif or the p-loop with a consensus GxxxxGKS/T and, PM2 and PM3 motifs, with conserved sequences T and DxxGQ, respectively, are strictly conserved in all three lineages of *Mimiviridae* (Figure 2A). The G1, G2, and G4 motifs, with consensus F, ANKxD, N, respectively are conserved, while the G3 motif is represented by either SSF or NCI (Figure 2A). *Mimiviridae* Rab GTPase possess all the conserved five Rab-family specific motifs, RabF1-RabF5, clustered around the switch regions I and II (Figure 2A). The first motif RabF1, represented by a consensus IGAAF in the *Mimiviridae* family, is critical for facilitating the crosstalk between the switch I and switch II residues (Dumas et al., 1999). The other four motifs, RabF2-RabF5, although show some variations, are readily identifiable and are present in the same structural context, between β3 and β4 sheets, and are closer to or part of the switch II region as in the well-known Rabs (Figure 2A) (Pereira-Leal and Seabra, 2000). Furthermore, the hypervariable region of *Mimiviridae* Rab is situated at the carboxy-terminal to the GTPase-fold that is followed by the CAAX boxes (C, cys; A, aliphatic; X, any amino acid). The CAAX boxes are the signature prenylation motif of Rabs that consists of two cysteine residues within the five residue stretch at the extreme C-terminus in one of the following combinations, XXXCC, XXCCX, XCCXX, CCXXX, or XXCXC (Pereira-Leal and Seabra, 2000). In *Mimiviridae* Rabs, the prenylation motif shows dimorphism as XXXCC and XXCXC (Figure 2A). Rab GTPases undergo prenylation through the attachment of geranylgeranyl moieties to these two cysteine residues that regulate the membrane insertion (Gomes et al., 2003).

Rab subfamilies are further distinguished based on the presence of subfamily specific conserved sequences (RabSF1-4) that show higher homology within the subfamily (Moore et al., 1995). The RabSF1-4 regions in the *Mimiviridae* family are also identified corresponding to sequences upstream of PM1, α1/loop3, α3/loop7 and α5, respectively (Figure 2A). To delineate the isoform-type of *Mimiviridae* Rabs that could give clues about their function, an alignment of only SF regions of Rab isoforms A–C with *Mimiviridae* Rabs was generated (Figure 2B). While the key residues in the SF1 and SF3 regions of *Mimiviridae* Rabs and Rab isoforms A–C are conserved, the residues in the SF2 and SF4 regions showed higher divergence from all other Rab5 isoforms (Figure 2B).

### Predicted structure of mimiviral Rab

The GTPase-fold consisting of five α-helices flanking a six-stranded β-sheet, five parallel and one antiparallel, is a common feature of the Ras superfamily (Dumas et al., 1999). These structural features were also identified in the predicted APMV Rab GTPase structure (Figure 2C). Motifs responsible for binding GTP and Mg^2+^ are located in the loop regions between α-helix and β-strands as seen in all the Ras superfamily proteins. The nucleotide-dependent Rab functions are primarily determined by the switch I and II regions. Solved structures indicate that the GDP bound state tends to have switch regions disordered, which upon binding to GTP, transmutes into a well-ordered structure (Stroupe and Brunger, 2000). The γ-phosphate of GTP forms contact with switch I and II regions (Lee et al., 2009). The switch I and II regions in the predicted APMV Rab structure are located in the loop2 and loop4-α2-loop5, respectively (Figure 2A).

### Mimiviral Rab GTPase has diverged early from lower eukaryotic Rab5 and belongs to group II family of Rab GTPases

**Figure 3:**
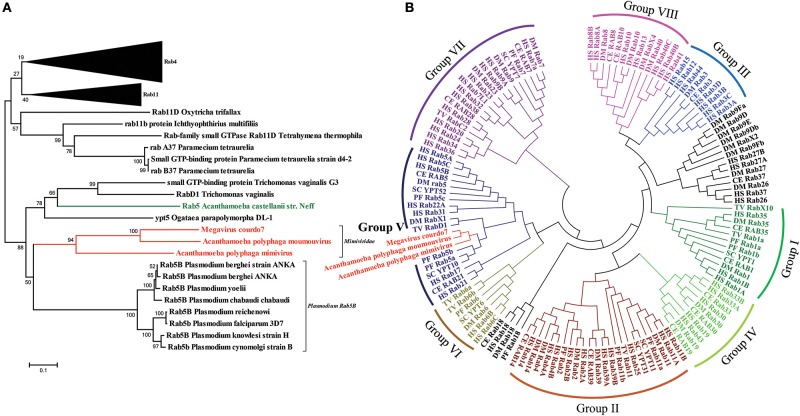
**Phylogenetic analysis**. **(A)** Neighbor-Joining phylogenetic tree of the amino acid sequences obtained through BLASTP results using APMV Rab as a query. *Mimivridae* family sequences are highlighted in red and *Acanthamoeba castellani str.Neff* sequence is highlighted in green. **(B)** Neighbor-Joining phylogenetic tree constructed from a dataset comprising of the amino acid sequences of the full Rab sequences of the following representative organisms: *Homo sapiens* (HS), *Plasmodium falciparum* (PF), *Caenorhabditis elegans* (CE), *Drosophila melanogaster* (DM), *Trichomonas vaginalis* (TV), *Saccharomyces cerevisiae* (SC). Group I Rabs are shown in green, Group II in maroon, Group III in blue, Group IV in lime green, Group V in navy blue, Group VI in olive green, Group VII in purple, Group VIII in pink and unclassified Rabs are shown black, and viral sequences are shown in red.

Homology search using APMV Rab GTPase retrieved Rab homologs in eukaryotes ranging from unicellular *Paramecium* to multicellular organisms like *Homo sapiens* and, different Rab families like Rab5, Rab11, and Rab4. A neighbor-joining phylogenetic tree of *Mimiviridae* Rab with homologs showed their close association with the lower eukaryotic Rabs, specifically, protozoan Rab GTPases, including a *Acanthamoeba castellanii str. Neff* Rab (Figure 3A). This analysis is consistent with an earlier report that suggested that the APMV R214 gene is closely related to a homolog found in its host amoeba (Moreira and Brochier-Armanet, 2008). Furthermore, the phylogeny showed two distinct clades; one consisting of *Mimiviridae* family Rab, *Plasmodium* Rab5B, *Acanthamoeba castellanii str. Neff* Rab5, *Trichomonas* RabD1; and the other consisting of Rab4 and Rab11 from a diverse group of organisms (Figure 3A). To further extend this analysis, all annotated Rab sequences from few representative organisms including *Mimiviridae* Rabs were used to generate a comprehensive phylogenetic tree and found that Rabs from *Mimiviridae, Plasmodium* and few lower eukaryotes form a separate clade (Figure 3B).

### Interaction network

**Figure 4:**
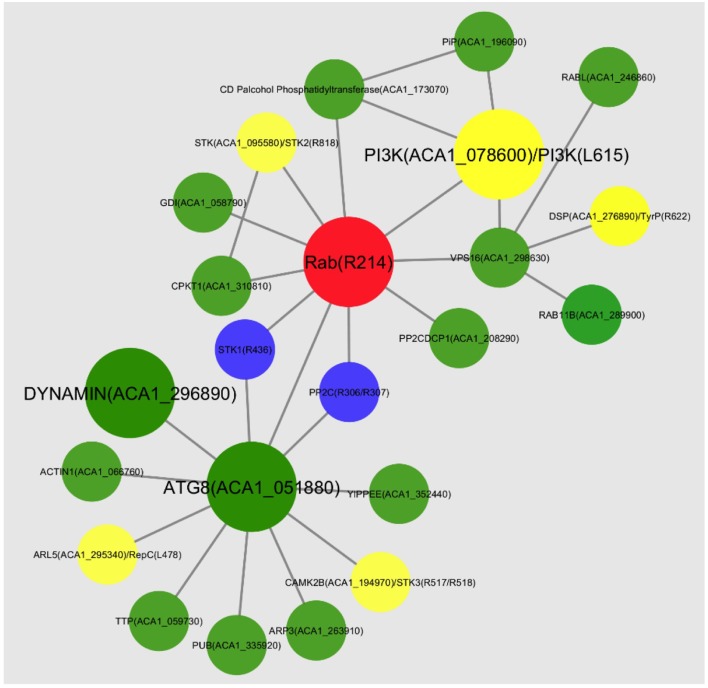
**Predicted interaction network of the APMV Rab GTPase**. *Pf* Rab5b template based interaction network of Mimivirus Rab (red) physical interactome. The host proteins that are recruited are shown in green, the proteins of viral origin are shown in blue, and the proteins that could be of either host or viral origin are in yellow. The key interacting partners discussed in the text are highlighted with a bigger font.

Interestingly, APMV Rab and *Plasmodium falciparum* Rab5B (*Pf* Rab5) are homologs and the phylogeny suggests that they share a common ancestor (Figure 3A). The physical interactome of *Plasmodium* Rab5B was constructed using the yeast interaction network as a template and the predicted interaction network was experimentally validated (Rached et al., 2012). For example, one of the proteins that was predicted, a *Plasmodium falciparum* coded casein kinase1 (*Pf* CK1), showed interaction with only *Pf* Rab5B, but not with other *Pf* Rab5 isoforms (Rached et al., 2012). We constructed the global physical interactome of APMV Rab GTPase using *Plasmodium* Rab5B interaction network as a template (Figure 4). The interactome predicted interacting partners from both APMV and its host Acanthamoeba. Some of the important interactors identified are autophagy-related protein 8 (Atg-8), dynamin (both are host factors) and phosphoinositide-3-kinase (PI3K), coded by APMV (Figure 4). We speculate that these proteins, along with Rab GTPase, are the key players of membrane remodeling during APMV capsid assembly and their probable functions are discussed below.

#### Atg8: an important player for membrane enlargement

Atg-8 is a ubiquitin-like protein that tethers to the membrane through its interaction with phosphatidylethanolamine (PE) (Nakatogawa et al., 2007). Atg-8 is most likely to be recruited from the host as it was not found in the APMV genome. The membrane-tethering and hemi-fusion activities of Atg-8 are pivotal for membrane expansion (Nakatogawa et al., 2007). Atg-8 also plays an important role in the enlargement of autophagosomal membranes during autophagosome formation (Mizushima et al., 1998; Ichimura et al., 2000).

#### Dynamin: helical scissors for vesicle scission

Dynamin, another protein recruited from the host, plays an important role during budding of vesicles from the Trans-Golgi Network (TGN) and endosomes (Jones et al., 1998; Kreitzer et al., 2000). Dynamin family members bind to and oligomerize helically around inositol lipid molecules and impart a tubular membrane topology (Hinshaw and Schmid, 1995; Marks et al., 2001). Cooperative recruitment of dynamin and BAR (Bin-Amphiphysin-Rvs) domain proteins induces positive curvature on the membrane with the help of exoskeleton/endoskeleton during vesicle scission (McMahon and Gallop, 2005).

#### Class III PI3K or Vps34: a component of the autophagosome vesicle formation complex

A Class III PI3K (phosphoinositide 3-kinase) or Vps34 is a kinase recruited to the vesicles in cells expressing active Rab5 and was found to colocalize with Rab5 bound to the endosomal tethering protein EEV1 (Christoforidis et al., 1999; Murray et al., 2002). Our genome-wide search found a PI3K homolog in APMV coded by L615 gene that could interact with APMV Rab and bring about vesicle enlargement. Rab5 has also been shown to form a complex with Vps34 and Beclin1 proteins that is essential for autophagosome formation (Ravikumar et al., 2008).

## Discussion

The NCLDV is a large, apparently monophyletic clade of viruses that consists of seven families of eukaryotic double stranded DNA viruses, namely, *Poxviridae, Iridoviridae, Ascoviridae, Asfaviridae, Phycodnaviridae, Marseilleviridae*, and *Mimiviridae* (Iyer et al., 2001, 2006; Yutin and Koonin, 2009). Based on the phylogenetic reconstructions of the highly conserved genes called as NCLDV clusters of orthologous groups of proteins (NCVOGs) which includes primase-helicase, DNA polymerase B, packaging ATPase and A2L-like transcription factor, *Mimiviridae* family has been subgrouped into two groups, I and II, and, group I *Mimiviridae* has been further delineated into three lineages; A, B, and C (Colson et al., 2012). *Mimiviridae* group I viruses possess a membrane layer underlining the icosahedral capsid (Xiao et al., 2005; Mutsafi et al., 2013). Source of this lipid bilayer and how the viruses acquire the membrane layer from endoplasmic reticulum cisternae have been demonstrated microscopically, although their molecular mechanisms remain unclear (Mutsafi et al., 2013).

All three lineages of group I *Mimiviridae* viruses' code for Rab GTPase (Figure 1A). Our thorough sequence analysis of *Mimiviridae* Rabs and the predicted structure of APMV R214 gene product suggests the presence of all the Rab signature motifs viz. RabF(1-5), RabSF(1-4), switch regions I and II, and the classical GTP binding motifs (Figures 2A,C). The clustering of all members of *Mimiviridae* under the same clade, sharing a common origin with the lower eukaryotes, suggests the acquisition of Rab GTPase by a *Mimiviridae* ancestor from a lower eukaryotic ancestor, probably through HGT events (Figure 3A). Furthermore, a comprehensive phylogenetic analysis with all annotated Rab subfamilies indicated the close homology of *Mimiviridae* Rabs to the lower eukaryotic Rab5B (Figure 3B). Based on the conservation pattern in RabSF regions, Rab5 subfamily has been further divided into three isoforms, namely, 5A, 5B, and 5C (Bucci et al., 1995). Our sequence analysis showed that *Mimiviridae* Rabs have significant sequence divergence in the RabSF regions. This is particularly interesting since RabSF2 residues are part of the switch region I, where the nucleotide-dependent conformational changes occur (Stenmark and Olkkonen, 2001). Because the switch region residues are exposed on the surface of the molecule, it was hypothesized that they are involved in binding to effectors and regulators (Stenmark and Olkkonen, 2001). Since Rab GTPases from different species cosegregate during phylogenetic analysis, it was suggested to use phylogenetic analysis as one of the criteria, along with specific sequence in the RabSF regions, to assign Rab subfamily (Pereira-Leal and Seabra, 2001). Divergence in the RabSF region in *Mimiviridae* Rabs implies different functional specificity dictated by specific effector/s and regulator/s suggesting that the *Mimiviridae* Rabs could potentially form a novel subfamily of Rab5, Rab5D. Apparently, Rab group V representative, Rab5, has a complex evolutionary history (Klöpper et al., 2012). It was suggested that the Rab5 duplication has occurred independently in fungi (Ypt52), apicomplexans (Rab5B), and kinetoplastids (Rab5B) (Klöpper et al., 2012). *Mimiviridae* Rab BLAST search failed to retrieve other isoforms of *Plasmodium* Rab5 viz. A and C, but selectively retrieves only Rab5B. This observation leads to the scenario that the acquisition of Rab by *Mimiviridae* family might have occurred concomitantly with the duplication event in the apicomplexans and evolved to be a new isoform Rab5D.

Interestingly, superimposition of Human Rab5B bound to GDP and the predicted APMV Rab structures, although showed high structural conservation with an RMSD (Root Mean Square Deviation) value of 1.08, the observed heterogeneity is localized in the switch regions (data not shown). The structural differences in the switch regions are the determinants of specificity toward effectors (Pfeffer, 2005; Lee et al., 2009). This observation is consistent with the RabSF sequence alignments and suggests divergence of an acquired Rab5B in the *Mimiviridae* family into a new isoform.

Although all known Rab5 isoforms are specialized in endosome fusion, some of them are also involved in endocytic function, endosome sorting etc. (Woodman, 2000). Studies have also shown that, besides the hypervariable region, certain F and SF regions are also important determinants of membrane targeting and effector protein interaction specificity (Ali and Seabra, 2005). The R214 transcript is not detectable at 3 h PI and is expressed optimally between 6 and 9 h PI (Legendre et al., 2011). In addition, proteomic study of the purified APMV virion particles showed that both Rab GTPase and PI3K homologs are not associated with the viral particles suggesting that their participation in the early events in establishing infection such as intracellular transport following phagocytosis is unlikely (Renesto et al., 2006). Earlier it was suggested that APMV Rab could be involved in the regulation of host cell cycle (Moreira and Brochier-Armanet, 2008). Here, we speculate that Rab GTPase could also play a role in the viral membrane acquisition during capsid assembly.

### A hypothetical model for membrane acquisition during mimivirus capsid assembly

**Figure 5:**
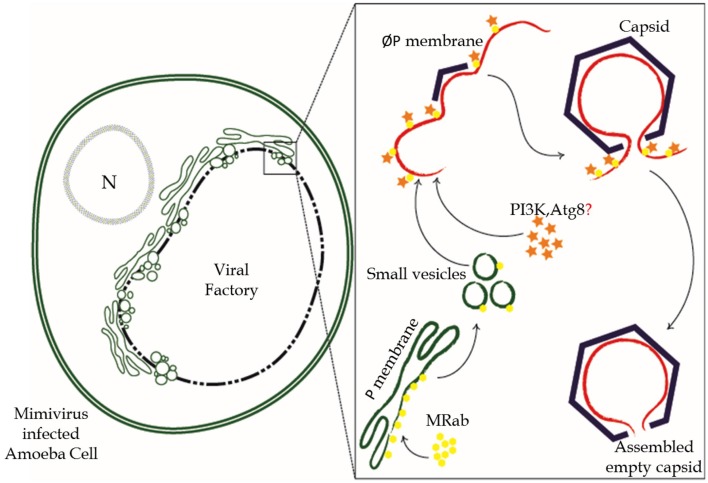
**Hypothetical model for the inner membrane acquisition during APMV capsid assembly**. The molecular events leading to the inner membrane acquisition for APMV capsid assembly begins with the expression of the Rab GTPase (yellow) at around 6 h PI. The ER and TGN membrane (green) cisternae gathered at the viral production lines of VF are then subjected to the Rab GTPase mediated pinching of small vesicles. These vesicles fuse together to form a membrane sheet with the help of Rab effectors like PI3K and Atg-8 (orange). The membrane, made amenable through de-phosphorylation (red), serves as a platform for the scaffolding protein and capsid assembly (violet).

The APMV Rab GTPase R214 gene expression starts at 6 h PI with highest expression at 9 h PI (Legendre et al., 2011). Microscopic studies indicated the appearance of viral factory at around 4 h PI (Suzan-Monti et al., 2007; Mutsafi et al., 2010). The presence of vesicular structures and membrane sheets on the periphery of the viral factory with extensive membrane network are seen at 7.5 h PI (Mutsafi et al., 2013). The small vesicles budding from the ER fuse together to form a multivesicular body (Mutsafi et al., 2013). On the basis of our interaction network analysis, we speculate that the Trans-Golgi network (TGN) could also contribute to the vesicles. The APMV Rab, which is optimally expressed at around the same time, could insert itself into the membrane through its C-terminal prenylation site, get localized near ER and/or TGN and initiate budding of small vesicles (Figure 5). APMV Rab could also facilitate the fusion of smaller vesicles by forming complex with Vps34 and Beclin1. Both Rab5 and Vps34 regulate Atg5-Atg12 conjugation and promote fusion of Atg5-rich phagosome leading to the formation of autophagosomes (Ravikumar et al., 2008). Further, Rab5 mediated recruitment of Atg-8 that is tethered to the membrane, leads to the hemifusion of small vesicles and expansion of autophagosomes (Nakatogawa et al., 2007). We speculate that the multivesicular bodies observed (Mutsafi et al., 2013) are the result of Atg-8 mediated fusion of smaller vesicles. These multivesicular bodies fuse and, then rupture leading to the formation of open sheets that are incorporated into the virion. The factors governing the rupture event and further stabilization of the ruptured membrane are yet to be identified (Mutsafi et al., 2013). The Rab GTPase, still bound to the membrane sheet, possibly recruits inositol-5-phosphatase that de-phosphorylates PI(4,5)P_2_ of the membrane (Sarantis et al., 2012). The de-phosphorylated membrane is amiable to be molded into any structural form and the scaffolding proteins initiate the expansion of the capsid angular structure over the “softened” membrane (Figure 5) (Chang-Ileto et al., 2011).

The presence of a membrane overhang, lining the assembled nascent capsid, was suggested to prevent the premature sealing of the capsid (Mutsafi et al., 2013). The membrane hang is trimmed after the completion of capsid assembly, leaving a ~20 nm non-vertex opening (portal) for genome packaging (Mutsafi et al., 2013). The host dynamin protein could mediate the membrane trimming by forming helical oligomers around the inositol lipid giving a tubular morphology to bring about scission (Figure 5, McMahon and Gallop, 2005).

## Conclusions

Our thorough sequence, structural, and phylogenetic analysis showed that the *Mimiviridae* coded Rab GTPases could constitute a novel isoform Rab5D. In addition, generating an interaction network helped in identifying potential viral and host proteins that might play a role APMV membrane biogenesis. Proposed hypothetical model for APMV membrane biogenesis suggests that intricate interactions between the viral Rab GTPase, other viral coded proteins and several host factors are necessary to bring about APMV membrane biogenesis. These insights gained from the *in silico* analysis will further aid in understanding the roles of viral and host proteins in APMV membrane biogenesis.

### Conflict of interest statement

The authors declare that the research was conducted in the absence of any commercial or financial relationships that could be construed as a potential conflict of interest.

## Appendix

### Accession numbers

Figure 1:

gi|11931713|162849299, gi|659666264, gi|392056049, gi|311977705, gi|557951747, gi|371943930, gi|363540521, gi|448825558, gi|371944856, gi|441432435, gi|310831487, gi|190359301, gi|585299378, gi|211956319, gi|325557805, gi|160857947.

Figure 3A:

gi|82050818: *Acanthamoeba polyphaga mimivirus*, gi|441432178: *Acanthamoeba polyphaga moumouvirus*, gi|371943588: *Megavirus courdo*7, gi|123471026: *Trichomonas vaginalis* G3, gi|471227609: *Ichthyophthirius multifiliis*, gi|403370178: *Oxytricha trifallax*, gi|159024328: *Aiptasia pulchella*, gi|62736284: *Trichomonas vaginalis*, gi|225711860: *Lepeophtheirus salmonis*, gi|358338265: *Clonorchis sinensis*, gi|511004806: *Mucor circinelloides* f. circinelloides 1006PhL, gi|31745716: *Toxoplasma gondii*, gi|675134504: *Hammondia hammondi*, gi|641579338: *Plasmodium reichenowi*, gi|124512956: *Plasmodium falciparum* 3D7, gi|470308728: *Capsaspora owczarzaki* ATCC 30864, gi|457876654: *Plasmodium cynomolgi* strain B, gi|672186478: *Plasmodium inui* San Antonio 1, gi|15986733: *Mus musculus*, gi|168001170: *Physcomitrella patens*, gi|221060859: *Plasmodium knowlesi* strain H, gi|307777770: *Tetrahymena thermophila*, gi|118355342: *Tetrahymena thermophila*, gi|168056370: *Physcomitrella patens*, gi|353232500: *Schistosoma mansoni*, gi|54792729: *Canis lupus* familiaris, gi|33150586: *Homo sapiens*, gi|675122722: *Hammondia hammondi*, gi|8394136: *Rattus norvegicus*, gi|148692254: *Mus musculus*, gi|431920194: *Pteropus alecto*, gi|237831345: *Toxoplasma gondii* ME49, gi|401407536: *Neospora caninum* Liverpool, gi|221061061: *Plasmodium knowlesi* strain H, gi|156102877: *Plasmodium vivax* Sal-1, gi|318270757: *Ictalurus punctatus*, gi|457876942: *Plasmodium cynomolgi* strain B, gi|74834378: *Paramecium tetraurelia*, gi|325190342: *Albugo laibachii* Nc14, gi|242021638: *Pediculus humanus* corporis, gi|74834500: *Paramecium tetraurelia*, gi|168062057: *Physcomitrella patens*, gi|241643394: *Ixodes scapularis*, gi|557240148: *Eimeria maxima*, gi|294895479: *Perkinsus marinus* ATCC 50983, gi|118372409: *Tetrahymena thermophila*, gi|57524538: *Danio rerio*, gi|646712526: *Zootermopsis nevadensis*, gi|557230549: *Eimeria necatrix*, gi|729714020: *Rhizopus microsporus*, gi|577151129: *Plasmodium vinckei* petteri, gi|124513178: *Plasmodium falciparum* 3D7, gi|225717876: *Caligus clemensi*, gi|225710156: *Caligus rogercresseyi*, gi|672186872: *Plasmodium inui* San Antonio 1, gi|562974055: *Ogataea parapolymorpha* DL-1, gi|675234676: *Plasmodium yoelii*, gi|661187471: *Lichtheimia corymbifera* JMRC:FSU:9682, gi|661186371: *Lichtheimia corymbifera* JMRC:FSU:9682, gi|74025730: *Trypanosoma brucei* brucei TREU927, gi|68075387: *Plasmodium berghei* strain ANKA, gi|749164803: *Gregarina niphandrodes*, gi|675228811:*Plasmodium berghei* ANKA, gi|703115059: *Morus notabilis*, gi|727139470: *Rhizopus microspores*, gi|444732045: *Tupaia chinensis*, gi|669196256: *Plasmodium vinckei* vinckei, gi|473785860: *Triticum urartu*, gi|675223050: *Plasmodium chabaudi* chabaudi, gi|294887922: *Perkinsus marinus* ATCC 50983, gi|167384556: *Entamoeba dispar* SAW760, gi|148922827: *Danio rerio*, gi|730370459: *Trichuris suis* gi|470391604: *Acanthamoeba castellanii* str. Neff, gi|357625222: *Danaus plexippus*, gi|124088730: *Paramecium tetraurelia* strain d4-2, gi|325191836: *Albugo laibachii* Nc14, gi|118387620: *Tetrahymena thermophila*, gi|224069898: *Populus trichocarpa*, gi|218204: *Oryza sativa* Japonica Group, gi|62897579: *Homo sapiens*, gi|669196060: *Plasmodium vinckei* vinckei, gi|89258415: *Suberites domuncula*, gi|528272994: *Angomonas deanei*, gi|557239031: *Eimeria necatrix*, gi|77404180: *Rattus norvegicus*, gi|62859319: Xenopus (Silurana) tropicalis, gi|67475925: *Entamoeba histolytica* HM-1:IMSS, gi|168040993: *Physcomitrella patens*.

Figure 3B:

*Homo sapiens* (HS)

sp|P62491, sp|P20340, sp|P51159, sp|Q13636, sp|Q15286, sp|Q86YS6, sp|Q9BZG1, sp|Q9NP90, sp|P61006, sp|P20338, sp|P20339, sp|P51151, sp|P51149, sp|P62820, sp|P61019, sp|Q9UL26, sp|Q14964, sp|Q96QF0, sp|Q9H0N0, sp|P51148, sp|O95716, sp|O14966, sp|Q15907, sp|Q9H0U4, sp|Q9ULC3, sp|P61106, sp|P51153, sp|Q9NRW1, sp|Q9UL25, sp|P61026, sp|Q9NP72, sp|Q6IQ22, sp|Q13637, sp|P61020, sp|P51157, sp|Q99P58, sp|A4D1S5, sp|Q969Q5, sp|Q96AH8, sp|P57729, sp|Q15771, sp|O95755, sp|P57735, sp|O00194, sp|P61018, sp|P20337, sp|Q8WUD1, sp|P20336, sp|Q92930, sp|Q9ULW5, sp|Q9H082, sp|Q9H0T7, sp|Q96DA2, sp|Q96AX2, sp|P59190, sp|Q96E17, sp|Q8WXH6, sp|Q96S21, sp|Q14088, sp|Q5JT25, sp|Q9NX57, sp|Q12829, sp|Q7Z6P3.

*Plasmodium falciparum* (PF)

tr|Q8I274, tr|Q7K6A8, tr|Q8IHR8, tr|Q76NM4, tr|Q7K6B0, tr|O96193, tr|Q8I5A9, tr|C0H5G2, tr|Q76NM7, tr|Q8I3W9, tr|C0H516.

*Caenorhabditis elegans* (CE)

sp|Q94986, sp|Q8MXS1, sp|P34213, sp|Q20365, sp|Q22782, tr|G5EFC1, tr|G4SEK4, tr|O01803, tr|P91857, tr|Q23146, tr|Q95QV3, tr|Q9U2C3, tr|Q1ZXR4, tr|Q93874, tr|Q9XWZ3, tr|Q22045, tr|Q18969, tr|Q9U1W9, tr|Q94148, tr|Q9UAQ6, tr|G5EFA6, tr|Q9XWR6, tr|Q6BCV5.

*Drosophila melanogaster* (DM)

sp|Q9VP48, sp|P25228, tr|Q9V3I2, tr|O76742, tr|O18338, tr|O18339, tr|Q7KY04, tr|Q9VM50, tr|O18336, tr|O18332, tr|Q9W3Q0, tr|Q9W585, tr|Q9W5X0, tr|Q8IPT6, tr|O18335, tr|O76901, tr|Q9W4A0, tr|Q9W2S9, tr|O18333, tr|Q7PLE9, tr|O18334, tr|O15971, tr|Q86BK7, tr|Q86BK8, tr|Q9VIW6, tr|Q8IR98, tr|Q9VNG6, tr|Q8IR80, tr|F0JAF7, tr|Q7PLE8, tr|Q8IRK9, tr|Q8IRK8, tr|Q9VYM8, tr|Q9VZ47, tr|Q95RQ2, tr|Q8SYS5, tr|Q9V3C2, tr|M9NEE8, tr|M9PG39, tr|O18337, tr|Q8T046, tr|Q95RH7, tr|B8A3X3, tr|Q8IGH2, tr|Q8MSX0, tr|Q8MRA4, tr|Q9VC31, tr|Q9W2V3.

*Trichomonas vaginalis* (TV)

tr|Q4FIE4, tr|Q4G290, tr|Q3YE89, tr|Q3YE90, tr|Q4G2B4, tr|Q4G2B7, tr|Q4G298.

*Saccharomyces cerevisiae* (SC)

sp|P01123, sp|P38555, sp|P51996, sp|P48559, sp|P36018, sp|P38146, sp|P32939, sp|Q99260.
